# Hemangioma of the Tympanic Membrane: A Case and a Review of the Literature

**DOI:** 10.1155/2012/402630

**Published:** 2012-12-02

**Authors:** Emilio Mevio, Marco Cazzaniga, Mauro Mullace, Donatella Paolotti

**Affiliations:** ^1^Department of Otorhinolaryngology, Ospedale Fornaroli, Via Donatore del Sangue, 20013 Magenta, Italy; ^2^Department of Pathology, Ospedale Fornaroli, Via Donatore del Sangue, 20013 Magenta, Italy

## Abstract

Hemangiomas of the external auditory canal, involving the posterior bony canal and the adjacent tympanic membrane, although rare, are considered a specific disease entity of the human external auditory canal. Hemangiomas of the tympanic membrane and/or external auditory canal are rare entities; there are 16 previous case reports in the literature. It is a benign vascular tumor. It generally occurs in males in the sixth decade of life. Total surgical excision with or without tympanic membrane grafting appears to be effective in the removal of this benign neoplasm. The authors present a case and a review of the literature discussing diagnostic and surgical approaches.

## 1. Introduction

Hemangiomas are benign vascular lesions that are most common in infancy and childhood. The tumor occurs frequently in head and neck. One third of these lesions will present at birth. Besides, 20% of hemangiomas are multiple. Hemangiomas of the tympanic membrane and/or external auditory canal are rare entities, with 16 previous case reports in the literature. It is a benign vascular tumor. It generally occurs in males in the sixth decade of life. It appears as a small vascular lesion interesting the deep posterior bony external auditory canal and/or the posterior superior tympanic membrane. The first two cases of hemangioma of the tympanic membrane and posterior external ear canal were reported by Freedman et al. in 1972 [1]. Balkany et al. in 1978 reported the first case of capillary hemangioma exclusively envolving the tympanic membrane [2]. In 1982, there was proposed a hemangiomas classification that divides these lesions in two histological categories: cavernous and capillary. The cavernous hemangioma is characterized by large cavernous vascular space lined by endothelium. Capillary hemangioma is made up of small vessel of capillary caliber. It is lobulated and lacks a capsule. In 1990, one case of mixed hemangioma (cavernous/capillary) has been described [3].

We present a case and a review of the literature discussing diagnostic and surgical approaches.

## 2. Case Report

A 55-year-old man presented with a 3-month history of left otalgia, hypoacusia, and pulsatile omolateral tinnitus.

**Figure 1:**
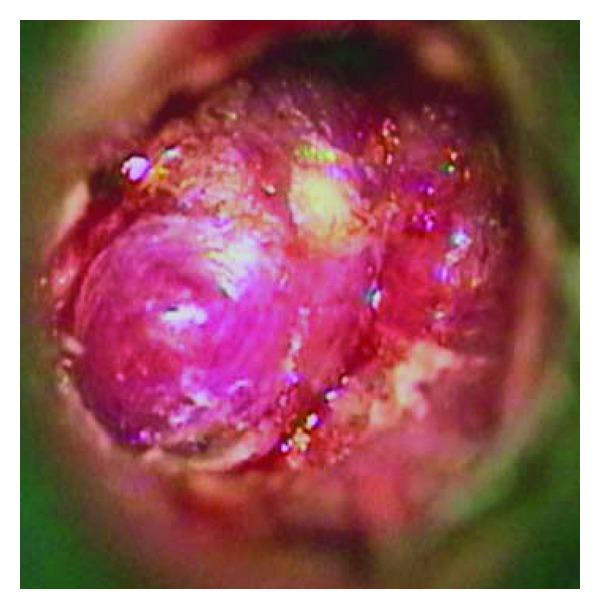
Otomicroscopic view of left tympanic membrane. The exofitic and vascular mass is seen in medial-superior part of the drum.

The otoscopic examination revealed a purple exophytic ovoid nonpulsatile lesion of the superior quadrant. It had pedicle in correspondence of malleus's manubrium (Figure 1).

**Figure 2:**
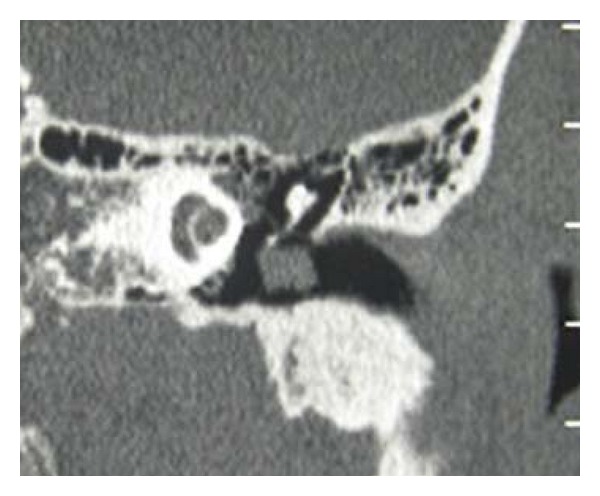
Computed tomography scanning without means of contrast. A 3 mm soft tissue mass is localized in the superior part of ear drum in correspondence of malleus with no present signs of erosion. Middle ear cavity and the other ossicles appeared normal.

A CT scan showed a round smooth mass (3 mm of diameter) external to the tympanic membrane (Figure 2). There was no evidence of bone erosion or middle ear extension. Audiometric testing showed mixed hearing loss in the left ear (20–30 dB air-bone gap at 250–1000 Hz).

A transcanal surgical resection was performed. The lesion was removed, leaving the fibrous and the mucosal drum layers intact.

**Figure 3:**
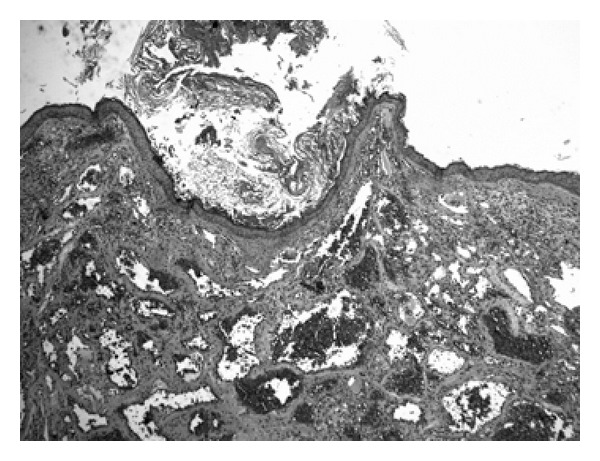
Cavernous hemangioma, HE, 50x. Histologic image of the tumours consisting in a well-defined proliferation of dilated tortuous vascular structures, closely adhering to each other, filled by erythrocytes in the lumen. They varied in size and were delimited by a single layer of flat endothelial cells. Superficially, the lesion was lined by hyperkeratotic squamous epithelium.

Pathologic examination revealed a well-defined proliferation of dilated tortuous vascular structures, closely adhering to each other, filled by erythrocytes in the lumen. They varied in size and were delimited by a single layer of flat endothelial cells. Superficially, the lesion was lined by hyperkeratotic squamous epithelium. These features were consistent with a cavernous hemangioma (Figure 3). The postoperative course was uneventful, and conductive hearing loss disappeared. There has been no recurrence for 1 year postoperatively.

## 3. Discussion

Hemangiomas involving the tympanic membrane (TM) and/or the adjacent posterior bony external auditory canal (EAC), although rare, do appear to be a specific disease entity of the human external auditory canal. Till now in the literature, there have been 17 reported cases of this disease (Table 1). The hemangioma of the TM and/or the EAC occurs predominantly in adulthood (average age 56), with a predilection for the male sex (M : F, 2 : 1).

Tympanic hemangiomas, 40% of external ear hemangiomas, arise from the lamina propria of the TM and has pedicles in the posterior part of the tympanic membrane [2, 4, 8, 11, 13, 14]. The hemangioma may affect the EAC involving only the skin of the posterosuperior wall (20% of cases) [6, 9, 10, 15]. It can sometimes extend to the adjacent tympanic membrane (40% of cases) [1, 3, 5, 7, 12]. Typically, the ear hemangiomas do not have bone invasion features. Only Jackson has described a case of mixed hemangioma envolving the bone of EAC [3].

From the histological point of view, in the overwhelming majority of cases, these neoplasms appear as cavernous hemangiomas (13 out of 17 cases). Capillary haemangiomas are rare (3 out of 17 cases) [2, 11, 13], and only one case of mixed hemangioma (cavernous/capillary) was reported [3].

The hemangioma may be incidentally found, not accompanied by any symptomatology, whether interesting only the TM or extending to the EAC (40% of cases). Sometimes, it is accompanied by rather nonspecific otologic symptoms: hearing loss, fullness, tinnitus, and otorrhea.

Differential diagnosis includes glomus jugulare or tympanicum, high jugular bulb, aberrant internal carotid artery, and arteriovenous malformation. In particular, the presence of bone erosion, the skull base relationship, and the middle ear status are essential in the aim of differential diagnosis. For this reason, temporal bone CT scan is considered the first choice to evaluate these entities. MRI is useful to carefully describe vascular lesions but does not provide any data regarding bony structures. For advanced lesions, an angiographic examination is advisable in order to plan an embolization before surgery [10].

The analysis of the cases, described so far, shows how hemangioma may occur either with exclusive involvement of the tympanic membrane, or EAC, or as a more extensive lesion affecting both sites. In the first situation, the surgical excision is relatively easy; we simply have to remove the external layer of the tympanic membrane, and rarely do we have to perform a myringoplasty in order to close a tympanic perforation [2]. In the case of lesion extended to the posterior wall of the ear canal, some authors have opted to extend the surgery even reaching the mastoidectomy [1, 5, 7].

All authors, except Magliulo, exclude the involvement of middle ear or mastoid. In fact, in no case histological examination after the surgical procedure showed involvement of the middle ear and/or mastoid. Even in the case described by Magliulo, hemangioma interested in full-thickness the tympanic membrane in correspondence of the handle of the hammer but without signs of erosion or pathology of the same [12]. In the light of the lack of aggressiveness of the disease, while in the past it tended to increase the area of excision and then use a myringoplasty, if not even to a exploratory mastoidectomy, the most recent reports indicate the simple excision technique of choice with respect to medial layers of the tympanic membrane. Recurrence of hemangioma has been reported only in one case and was related to inadequate surgical excision [3].

## Figures and Tables

**Table 1:** 

Author	Age	Sex	Location	Otologic symptom	Surgical approach	Pathology
Freedman, 1972 [1]	52	M	EAC + TM	None	Excision + Myringoplasty	Cavernous
Freedman, 1972 [1]	57	M	EAC + TM	None	Excision + Myringoplasty	Cavernous
Balkany, 1978 [2]	63	F	TM	None	Excision + Myringoplasty	Capillary
Andrade, 1983 [4]	59	M	TM	None	Excision + Myringoplasty	Cavernous
Kemink, 1983 [5]	52	M	EAC + TM	None	Excision + Mastoidectomy	Cavernous
Hawke, 1987 [6]	55	M	EAC	Otorrhea	Excision	Cavernous
Jackson, 1990 [3]	60	F	EAC + TM	HL	Biopsy + Excision	Mixed
Joshi, 1999 [7]	16	M	EAC + TM	Otorrhea	Excision + Mastoidectomy	Cavernous
Bijelic, 2001 [8]	78	F	TM	None	Excision + Myringoplasty	Cavernous
Reeck, 2002 [9]	53	M	EAC	HL + T	Excision	Cavernous
Limb, 2002 [10]	67	F	EAC	HL + T	Excision	Cavernous
Hiraumi, 2005 [11]	51	M	TM	Otorrhea + HL	Excision	Capillary
Magliulo, 2007 [12]	63	M	EAC + TM	HL + T	Excision + Myringoplasty	Cavernous
Spector, 2010 [13]	59	F	TM	HL + T	Excision	Capillary
Jang, 2011 [14]	49	M	TM	HL + T	Excision + Myringoplasty	Cavernous
Martines, 2012 [15]	59	M	EAC	HL + T	Excision	Cavernous
Mevio (this case)	55	M	TM	HL + T	Excision	Cavernous
